# Facilitators and Barriers to Recruiting Ambulatory Oncology Practices Into a Large Multisite Study: Mixed Methods Study

**DOI:** 10.2196/14476

**Published:** 2020-04-20

**Authors:** Milisa Manojlovich, Louise Bedard, Jennifer J Griggs, Michaella McBratnie, Kari Mendelsohn-Victor, Christopher R Friese

**Affiliations:** 1 School of Nursing University of Michigan Ann Arbor, MI United States; 2 Michigan Oncology Quality Consortium Ann Arbor, MI United States; 3 Hematology & Oncology Division Department of Internal Medicine University of Michigan Ann Arbor, MI United States

**Keywords:** recruitment activities, ambulatory care facilities, health services research

## Abstract

**Background:**

Practice-based research is essential to generate the data necessary to understand outcomes in ambulatory oncology care. Although there is an increased interest in studying ambulatory oncology care, given the rising patient volumes and complexity in those settings, little guidance is available on how best to recruit ambulatory oncology practices for research.

**Objective:**

This paper aimed to describe the facilitators and barriers to recruiting ambulatory oncology practices into a large multisite study.

**Methods:**

Using a mixed methods design, we sought to recruit 52 ambulatory oncology practices that have participated in a state-wide quality improvement collaborative for the quantitative phase. We used 4 domains of the Consolidated Framework for Implementation Research (CFIR) to describe facilitators and barriers to recruitment.

**Results:**

We successfully recruited 28 of the 52 collaborative-affiliated practices, collecting survey data from 2223 patients and 297 clinicians. *Intervention attributes* included multimodal outreach and training activities to assure high fidelity to the data collection protocol. The *implementation process* was enhanced through interactive training and practice-assigned champions responsible for data collection. *External context* attributes that facilitated practice recruitment included partnership with a quality improvement collaborative and the inclusion of a staff member from the collaborative in our team. Key opinion leaders within each practice who could identify challenges to participation and propose flexible solutions represented *internal context* attributes. We also reported lessons learned during the recruitment process, which included navigating diverse approaches to human subjects protection policies and understanding that recruitment could be a negotiated process that took longer than anticipated, among others.

**Conclusions:**

Our experience provides other researchers with challenges to anticipate and possible solutions for common issues. Using the CFIR as a guide, we identified numerous recruitment barriers and facilitators and devised strategies to enhance recruitment efforts. In conclusion, researchers and clinicians can partner effectively to design and implement research protocols that ultimately benefit patients who are increasingly seeking care in ambulatory practices.

## Introduction

### Background

A growing proportion of health care is delivered in ambulatory practice settings, yet little information about ambulatory care quality and safety is available [1]. Ambulatory practice settings are diverse in scope and oversight and include those embedded in hospitals and health systems as well as free-standing buildings, where private, individual, or group practices deliver care to ambulatory patients. Increasingly, substantial amounts of complex care are delivered in ambulatory practice settings, where research has an important role in improving the quality and safety of patient care [2]. Given the rising patient volumes and complexity, researchers have an increased interest in studying ambulatory practice settings for descriptive, interventional, and implementational research. Conducting research in ambulatory practice settings can be a daunting task, posing unique challenges to recruitment. Recruiting ambulatory practices requires obtaining endorsement from the practices themselves before recruiting individual participants who can be patients, health care providers, and other staff. Researchers have identified barriers and facilitators to the successful recruitment of community health centers [3] and primary care practices [4] for example, but specialty practices such as ambulatory practice settings that provide care to oncology patients may have other recruitment challenges, and we know little about those.

### Objectives

We sought to recruit ambulatory oncology practices that delivered chemotherapy to patients with cancer in our study. The purpose of our study was to understand health care delivery by characterizing clinician communication processes, communication technologies, and adverse patient outcomes in ambulatory oncology practices and to examine how these practices and technologies influence safe chemotherapy administration. Once practices agreed to participate, we recruited clinicians who worked in those practices and patients who were cared for at those sites. During the recruitment of practices into our study, we faced challenges mirroring what has already been reported in the literature [4-8]. For example, practice administrators frequently act as gatekeepers and make decisions about study participation on behalf of clinicians in the practice [4]. Clinicians have also reported difficulty in balancing the demands of research participation with patient care responsibilities [6].

Thus, the purpose of this paper was to report on the facilitators and barriers to the recruitment of ambulatory oncology practices in Michigan, United States, and share the lessons we learned.

## Methods

### Study Design

The overall study is using a mixed methods design. We began with a quantitative phase, by distributing questionnaires to all prescribers (ie, physicians, physician assistants, and nurse practitioners) and registered nurses who work in a sample of ambulatory oncology practices. In addition, for 6 weeks, site study coordinators completed a 1-page daily event log, and patients completed a 1-page self-reported symptom questionnaire. We then used the survey results to identify 8 practices for subsequent exploration via in-depth qualitative methods, and the analysis phase of the project is ongoing.

### Setting

The study setting includes ambulatory oncology practices that belong to the Michigan Oncology Quality Consortium (MOQC). MOQC is an alliance of ambulatory oncology practices formed with the purpose of sharing and benchmarking their data to improve the quality of oncology care. As we were interested in targeting ambulatory oncology practices throughout the state of Michigan in the United States, we partnered with MOQC, which currently has 52 affiliated practices all over Michigan.

### Procedures

We invited all MOQC-affiliated ambulatory oncology practices to participate in our study. We sought to recruit as many MOQC-affiliated practices as possible because we were interested in understanding the variation in clinician communication processes, communication technologies, and adverse patient outcomes in ambulatory oncology practices. The practices identified employees to serve as study coordinators who were responsible for distributing clinician questionnaires once, completing daily event logs, and distributing self-report questionnaires to patients daily for 6 weeks. Clinician questionnaires were about the usability of and satisfaction with the electronic medical record, communication among clinicians, perceptions of a safety climate, and perceptions of the work environment. The daily event logs summarized clinic activities and events related to chemotherapy (eg, the number of patients prescheduled and the number of patients who called the clinic for toxicity management). In the patient questionnaires, patients were asked to report symptoms related to their chemotherapy treatment. The survey procedures have been described elsewhere [9].

### Data Collection and Analysis

By collaborating with MOQC, we had access to the latest information about the various practices to use for recruitment purposes (eg, name and contact information of the practice manager). Practice recruitment occurred on a rolling basis from April 2017 to November 2017. During that time, we held weekly meetings, where we reviewed practice enrollment and survey response data. KV maintained a tracking sheet of all MOQC sites that had tabulated information of when sites were initially contacted, the dates of follow-up, and the identified reasons for nonparticipation. Overall, 2 research team members (MM and KV) reviewed the notes taken during these meetings to identify barriers and facilitators to recruitment. The entire research team met regularly to discuss and confirm emerging barriers and facilitators.

We used the Consolidated Framework for Implementation Research (CFIR) to help identify facilitators and adapt barriers to successful recruitment and implementation of data collection. We followed the example set by Coronado et al [3] who also used CFIR to organize barriers and facilitators to participation in their study. CFIR is an organizing framework that assesses potential factors that may influence implementation, grouping those factors into larger domains, which include intervention attributes, implementation process, external context, internal context, and characteristics of the individual involved [10]. We used all the CFIR domains, except for the characteristics of the individuals involved.

## Results

Using the CFIR framework, we successfully recruited 28 of the 52 MOQC-affiliated practices to participate in our study (a recruitment rate of almost 54%). From those participating practices, survey data were collected from 297 clinicians (a 68% response rate) and 2223 patients (a 58.7% response rate).

### Intervention Attributes

Intervention attributes refer to features of the intervention that can influence its execution, in our case, the characteristics surrounding data collection that could be customized to each practice without compromising the quality of data collected. Facilitators included involving physicians and ambulatory practice staff in the early stages of the project to get feedback on study materials and protocols. For example, 1 site developed a comprehensive, 1-page *helpful tips* sheet clarifying the data collection guidelines and patient eligibility criteria. The site gave us permission to distribute the tip sheet, acknowledging the original author, to all participating sites, and this also facilitated recruitment.

### Implementation Process

Implementation processes are the strategies that affect the implementation of interventions. Although we are not conducting an intervention as a part of our study, the implementation process refers to the training and use of staff for data collection. Each facility designated a *practice champion* who was a staff member responsible for overseeing data collection, assuring that data were being collected as scheduled, and notifying the research team of any barriers to data collection. Rather than trying to identify such an individual ourselves, we facilitated recruitment by asking participating practices to assign a practice champion, usually someone with discretionary time and workflow flexibility. Each champion’s primary role varied by the practice site. For example, in some sites, the practice manager acted as a champion and delegated data collection to a practice nurse, whereas in other practices, the nurse manager was the champion who collected all the data.

As we were asking sites to collect data from multiple sources, practice champions were required to attend a Web-based training session to learn about the study and assure that data would be collected uniformly at each site. Participating in the training was a mandatory requirement for engaging with us in the study, and training had to be completed before data collection could begin. We used PowerPoint slides (Microsoft Corporation, Redmond, Washington) [11] to present study information, and as the training was Web-based, we made it interactive by pausing during the presentation to ask for questions and input. We took the practice champions’ suggestions for customization to facilitate data collection at each site. We used questions provided by the champions during each training session to edit and enhance the slides to promote greater clarification in subsequent training sessions.

To further facilitate data collection, we scheduled 15- to 20-min telephone conversations with practice administrators and physician leaders to describe the study in greater detail. Before each scheduled conversation, the study information consisting of a single-page overview of the study and a *frequently asked questions* (FAQ) sheet was sent. We also produced a 2-min video showing a high-level overview of the study. The 2-min video was essentially a *talking head* of one of the investigators (MM) who highlighted the benefits of study participation. The video was taken with a cell phone, edited using Camtasia (TechSmith Corporation, Okemos, Michigan) [12], and uploaded onto our secure box site. We also prepared a separate PowerPoint presentation that we shared with practices that were considering participation, again highlighting the benefits of participation to practices. In a few cases, we conducted in-person, informational site visits and distributed the aforementioned supplemental materials.

### The External Context

The external context refers to environmental factors outside of each ambulatory practice, including MOQC practices throughout the state of Michigan and our efforts to recruit as many MOQC-affiliated practices as possible. Our recruitment efforts required several facilitative strategies, beginning with our presence at a MOQC biannual meeting that was attended by practice managers, physician leaders, and other MOQC stakeholders. The director of MOQC, who is a coinvestigator in the study, introduced the study to meeting attendees, highlighting how participation in the study would be relevant to the quality of care in individual practice settings. We provided attendees with a 1-page overview and study FAQ sheet. After the meeting, the MOQC director personally spoke with most physician leaders to remind them of the study and let them know that the study team would be contacting practices as a part of the recruitment process. In this way, the close professional relationships established by the director of MOQC with affiliated practices meant that we did not approach practices *cold*.

One of our earliest strategies to facilitate recruitment was to have a MOQC representative on the study team who was known to the practice staff. The MOQC representative served as a liaison to the MOQC director and as a point of contact for queries coming from MOQC-affiliated practices about the study. It was important that the study not fracture the practices’ pre-existing relationships with the MOQC office and the director; therefore, having a MOQC representative on the study team was essential.

### The Internal Context

The internal context refers to the characteristics of the implementing organization, specifically the unique characteristics and culture of each practice. Each practice differed both in terms of size and ownership as well as variation in patient populations served. The study team tailored procedures to the unique characteristics of each practice to facilitate recruitment. For example, some practices consisted of two or more physical locations. To develop a comprehensive understanding of the overall practice, our goal was to recruit all locations of a single practice into the study, although some smaller locations saw patients only 2 or 3 days a week. We treated each physical location as an individual unit in recognition of the unique culture of each location.

We also acknowledged that the practice staff were in the best position to advise us on data collection procedures. We had conversations with the practice staff to clarify the inclusion criteria for patients in our study because in some practices, noncancer patients were treated with antineoplastic agents. Clarifying that the drug had to be delivered to a patient with cancer made it easier for practices to determine which patients to include.

The practice administrators were often gatekeepers who allowed access to stakeholders within each site so as to facilitate access, and the MOQC representative on our team sent periodic study updates to the practice administrators about the number of participating practices without identifying specific sites. Although the institutional review board (IRB) of our institution deemed our study to be exempt from an ongoing IRB review, we learned that this was insufficient for many practices that required separate IRB determination from their own home institutions. Upon request, we shared with practices the study protocol developed for our IRB.

## Discussion

### Principal Findings

To our knowledge, we are the first to report on ambulatory oncology practice recruitment, as the research to date has focused on recruiting primary care practices. Recruiting practices for research requires multiple strategies to succeed, both at the practice and individual levels, no matter the specialty. By framing our recruitment strategies according to CFIR domains of intervention attributes, implementation process, external context, and internal context [10], we overcame potential barriers and applied facilitators to recruitment and data collection. We also learned several important lessons as a result of our recruitment efforts.

Coronado et al [3] offered choices and flexibility to primary care clinics participating in their study to facilitate intervention implementation. Similarly, we took the advice of our practice champions to adapt some of our methods to the workflow and resources used by the ambulatory practices. Better aligning the research and workflow methods helped build a rapport and facilitate engagement. For example, the practice champions told us that faxing was the easiest way to return data. Setting up a fax line was not a part of our original research plan, but it made the practices feel more like partners in the study. Being flexible with structural aspects of data collection to mirror practices’ processes enabled fluid implementation. The value of flexibility to practices’ traits during implementation research has been noted elsewhere [3,13].

In recruiting ambulatory practices, it is important to enlist the support of more than the medical director of the practice, as he or she may have limited time to devote to research or not necessarily be a *leader* of a practice [14]. In these instances, other practice staff may be in charge of the day-to-day activities and become responsible for fulfilling the research needs. Therefore, we enlisted practice champions to lead the data collection and research protocol implementation as a part of the implementation process. As the staff may feel resentful of the extra work for participating if they are not consulted from the beginning, we had practices self-assign a practice champion to give them some control in the research process, which was key to getting buy-in for the project [14]. Studies have reported that a frequently mentioned reason for nonparticipation was not having enough time to engage in research studies or difficulties allocating staff for the research [4,7]. To address this potential barrier, we provided training and accessible support to those practice champions, which had the additional benefit of overcoming difficulties associated with incorporating and following study protocols [7].

A central facilitator that affected the external context focused on our collaboration with MOQC. There were many advantages to the collaboration that facilitated recruitment, including access to an established infrastructure and the latest information about each of the practices in the consortium to facilitate recruitment. Johnston et al [8] found that the lack of latest information was a tremendous barrier to recruiting primary care practices because of the additional time and effort researchers needed to invest to get that information. Another advantage of collaborating with MOQC was having a MOQC representative on the study team who could attend weekly meetings, a pivotal recruitment strategy. This is consistent with recommendations in the guidelines developed for researchers interested in conducting clinical trials in practice-based research networks [5] but has not been reported previously in ambulatory oncology research. We used the pre-existing relationship between the MOQC representative and the practices to increase the likelihood of getting favorable responses. Typically, unless a MOQC representative made the initial contact, the sites were either unwilling to talk with the study team members or denied knowledge of the study.

During the MOQC biannual meeting, and in individual phone calls, the MOQC director was careful to highlight how participation in the study would be relevant to the quality of care in individual practice settings to address barriers in the internal context. The director of MOQC, as well as the MOQC representative mentioned earlier, had a positive influence on site participation because of the professional networks built with physicians over the years. Such alliances are the foundation of practice-based research networks, which operate as loose coalitions of primary care practices to improve clinical practice and patient outcomes [15]. Developing a relationship with an established practice-based research network or quality collaborative, as was done in this study, would facilitate recruitment in many types of ambulatory practices. In addition, the FAQ sheet provided information on the relevance of the research topic to physicians’ practices and monetary incentives. These strategies put the research into context for practices and align with research showing that distinguishing the individual benefits of research facilitates participation [3,16].

We also used many strategies to build a rapport with individual practices. Considering that practices function through the work of many people, it was important to build a rapport with a variety of providers and staff. Our early strategies for building a rapport focused on communication that frequently flowed through the practice administrator, receptionist, and support staff. This, along with having the MOQC representative on the study team, counteracted the inability to build a rapport with a practice’s receptionist, which has been identified as a barrier to recruitment [8]. In addition, by building a rapport with practice champions, providers, and medical directors, we were able to tap into multiple levels of leaders. Tapping into the power of opinion leaders or people considered to be likable, trustworthy, and influential has been shown to have a positive effect on promoting evidence-based practice, although the level of effectiveness does vary [17].

Another cited barrier to research participation is the lack of monetary incentives [7]. To overcome this, we offered a US $1000 incentive to each participating practice at the end of the 6-week data collection period, as an acknowledgment of the effort expended by the practice champion to collect data. The use of monetary incentives has been shown to increase the survey response rate compared with no use of incentives [18].

### Lessons Learned

We learned 2 important lessons related to distributing monetary incentives, which have not previously been reported. First, university policy required that a current W-9 be on file for each practice before incentive disbursement. A W-9 is a form used in the US income tax system to confirm information for income-generating purposes. Completing this paperwork, even though required by the Internal Revenue Service in the US, added to the overall burden, especially for smaller practices. As we did not have the information to complete a W-9 on their behalf, we used email and telephone prompts to encourage practices to complete the W-9 paperwork. The second lesson was related to communication about the incentives. As some practices were spread across multiple physical locations, each participating location was eligible for the incentive. However, as the university sent out incentives addressed to the practice and not each location, confusion arose when one practice called to ask why they had received an honorarium check. As a result, we intercepted the outgoing mail so that we could insert a thank you note and an explanation for the enclosed check, before returning the letter to the mail.

We learned another lesson through our challenges with getting an IRB approval from multiple sites and navigating a complex IRB system. Our experience may no longer be relevant in the near future, at least for research conducted in the United States, as US researchers will face new challenges because of the changes in the IRB process. Specifically, the Federal Policy for the Protection of Human Subjects (also known as the Common Rule) in the United States changed in January 2019 [19], and the National Institutes of Health (NIH) will require a single IRB submission to NIH for multisite research proposals starting in January 2020. The barrier of having to obtain an IRB approval from multiple sites will no longer exist in US-based research. No matter where the research is conducted, building sufficient time for an IRB approval into the timeline is a good strategy. A couple of weeks elapsed between recruitment and data collection at each practice because of training requirements for the practice champion. During this time, the IRB process could have been initiated had we asked about practice-specific IRB requirements. Communicating with practices about their own policies should occur early on to mitigate other challenges that can pose significant barriers to research participation.

Recruiting practices took longer than anticipated, providing us with a final lesson learned. Recruitment took 9 months, a time frame reported in other studies [8]. In many cases, we entered into negotiations with practices that were considering participation but had not yet made a final decision. We tried to address practice concerns by allowing practices to deal with competing priorities to allow them more time to engage with us. We extended the recruitment timeline for some practices that told us about other conflicts of time, such as a practice champion on leave, staff illnesses, or other obligations (all cited as barriers to participating in research [7]). The goodwill engendered by our flexibility contributed to practice engagement.

### Conclusions

Practice-based research in ambulatory care is essential to generate the data necessary to understand patterns of health care delivery, correlates, and outcomes in these diverse and understudied settings. Generating robust research data in ambulatory practice settings requires novel partnerships among researchers, coalitions, and a broad array of clinicians and practice administrators. Our experience provides other researchers with challenges to anticipate and possible solutions to common issues. Using CFIR as a guide, we devised strategies to facilitate recruitment efforts and minimize barriers. In conclusion, researchers and clinicians can partner effectively to design and implement research protocols that benefit not only researchers and providers but also the patients who are increasingly seeking care in ambulatory practices.

## Abbreviations

CFIR: Consolidated Framework for Implementation Research
FAQ: frequently asked questions
IRB: institutional review board
MOQC: Michigan Oncology Quality Consortium
NIH: National Institutes of Health
